# Roles of vasopressin in renal blood flow and vascular resistance in male mice after treadmill running: A mechanism for ALPE

**DOI:** 10.14814/phy2.70648

**Published:** 2025-12-11

**Authors:** Kazutoshi Nomura, Mamoru Tanida, Ryoko Akai, Tomohisa Yabe, Ai Fujii, Kanae Nomura, Keiichiro Okada, Kazuaki Okino, Norifumi Hayashi, Keiji Fujimoto, Yasutaka Kurata, Takao Iwawaki, Kengo Furuichi

**Affiliations:** ^1^ Department of Nephrology Kanazawa Medical University Uchinada Ishikawa Japan; ^2^ Department of Physiology II Kanazawa Medical University Uchinada Ishikawa Japan; ^3^ Division of Cell Medicine, Department of Life Science, Medical Research Institute Kanazawa Medical University Uchinada Ishikawa Japan

**Keywords:** acute kidney injury, exercise‐induced injury, in vivo imaging, pimonidazole, vasopressin

## Abstract

Acute renal failure with severe loin pain and patchy renal ischemia after high‐intensity exercise (ALPE) is a rare form of acute kidney injury. This study examined the role of vasopressin and its receptor, AVPR1A, in ALPE using a mouse model of brief high‐intensity treadmill exercise. Hypoxia‐reporter mice received vasopressin after treadmill running (Treadmill + Vasopressin group), whereas controls received vasopressin alone. Renal ischemia and hypoxia were assessed by in vivo imaging system (IVIS) after D‐luciferin injection and confirmed by pimonidazole staining. Renal blood flow and vascular resistance were measured in anesthetized mice. The effects of the AVPR1A antagonist SR49059 were also evaluated. The Treadmill + Vasopressin group showed a rapid decrease in renal blood flow and an increase in vascular resistance compared with the Vasopressin group. Pimonidazole staining revealed marked hypoxia at the corticomedullary junction, which was minimal in controls. Bioluminescence indicated renal hypoxia lasting up to 48 h. SR49059 attenuated these changes, supporting an AVPR1A‐mediated mechanism. These findings implicate vasopressin in the pathogenesis of ALPE via AVPR1A‐dependent vasoconstriction that leads to renal ischemia and support AVPR1A antagonism as a potential therapeutic strategy for ALPE.

## 1 INTRODUCTION

Acute renal failure with severe loin pain and patchy renal ischemia after high‐intensity exercise (ALPE) is an acute kidney injury syndrome that develops after brief intense exercise and is accompanied by severe loin pain (Ishikawa, 2002). First described by Ishikawa et al. in 1982 (Ishikawa, 1982), this syndrome and its diagnostic criteria are now widely accepted. ALPE predominantly affects young males (>90% in case series) (Ishikawa, 2002). Severe loin pain typically begins 3–12 h after exercise. Although the renal prognosis is generally good, the severity of pain necessitates differentiation from other conditions (Lee et al., 2012). Patients often experience nausea, vomiting, low‐grade fever, and elevated C‐reactive protein (CRP), leading to frequent misdiagnoses such as acute gastroenteritis or urolithiasis (Shoho & Kuriyama, 2021). The overall prevalence of ALPE remains unknown. Initially reported mainly in Asia, cases have since been documented in the United States, Europe, the Middle East, and New Zealand (Hughes & Miller, 2024; Kelly et al., 2021; Shoho & Kuriyama, 2021).

Patients with ALPE typically present with nonoliguric acute kidney injury and variable elevations in serum creatinine. ALPE is regarded as a distinct clinical entity characterized by nonoliguric acute kidney injury without significant rhabdomyolysis. Key differentiators are serum myoglobin and creatine kinase (CK) levels, which are normal or only slightly elevated. The precise pathogenesis remains unclear. However, wedge‐shaped areas of prolonged contrast retention on contrast‐enhanced CT suggest that heterogeneous renal vasoconstriction impairs blood flow and induces patchy ischemia. Ishikawa hypothesized that impaired perfusion, particularly at the level of the arcuate to interlobar arteries, results in insufficient washout of contrast medium, producing the characteristic wedge‐shaped appearance (Kelly et al., 2021). Although renal vascular tone is regulated by multiple hormones—including angiotensin, catecholamines, and vasopressin—the dominant factor(s) in ALPE are still unknown.

Vasopressin, synthesized in the hypothalamus and released from the posterior pituitary, is a potent vasoconstrictor that elevates blood pressure in settings such as hemorrhagic and septic shock. It acts on vascular smooth muscle via the arginine vasopressin receptor 1A (AVPR1A) (Guelinckx et al., 2016; Landry et al., 1997; Tsuneyoshi et al., 2005; Wisniewski et al., 2011). Vasopressin receptors comprise three subtypes—AVPR1A, AVPR1B, and AVPR2—all of which are G protein‐coupled receptors. AVPR1A is expressed in multiple organs, including the kidney, and within the kidney it localizes to arcuate and interlobular arteries (Haider et al., 2021; Hansen et al., 2005; Terada et al., 1993). Serum vasopressin increases with exercise (Röcker et al., 1989), particularly when intensity exceeds ~60% of maximal oxygen consumption (Freund et al., 1991), and this rise is relatively short‐lived (~1 h) (Röcker et al., 1989). In addition, nonspecific stressors associated with exercise (e.g., pain and emotional stress) can trigger vasopressin release (Robertson, 2006). Despite these observations, the clinical and physiological links between exercise‐induced vasopressin secretion and renal ischemia remain incompletely defined. Based on these findings, we hypothesized that exercise‐related vasopressin signaling contributes to ALPE. In the present study, we investigated the involvement of vasopressin and AVPR1A—particularly in the arcuate and interlobular arteries—in the pathogenesis of post‐exercise acute kidney injury using a mouse model of ALPE.

## 2 MATERIALS AND METHODS

### 2.1 Animals and ethical approval

Six‐ to eight‐week‐old male mice were used. Hypoxia‐reporter mice were heterozygous FVB.129S6‐Gt(ROSA)26Sor^tm2(hypoxia‐inducible factor 1‐alpha [HIF1A]/luciferase [Luc])Kael/J (oxygen‐dependent degradation [ODD]‐Luc; JAX #006206); age‐matched FVB/NJcl males (Nippon Clare Co., Tokyo, Japan) served as controls. Mice were housed in a temperature‐controlled room (24 ± 2°C) with a 12‐h light/dark cycle and had ad libitum access to water and a standard rodent diet (Lab MR Stock, Nosan Corporation, Yokohama, Japan). All procedures were approved by the Kanazawa Medical University Animal Experimentation Committee (Approval No. 2020‐34) and conducted in accordance with institutional guidelines. After experiments, mice were euthanized by cervical dislocation under a combination anesthetic as per facility practice.

### 2.2 Treadmill protocol (brief high‐intensity bout)

To model a brief high‐intensity stimulus, mice were acclimated to treadmill running (MK‐685, Muromachi Ltd., Tokyo, Japan) and then ran at 0° incline and 22 m/min for 1 min (Seldeen et al., 2019). Oxygen consumption was not measured; based on speed–VO_2_ relationships (Schefer & Talan, 1996) and maximal lactate steady‐state (MLSS) estimates around 20–22 m/min (Orsi et al., 2023), this workload was classified as high‐intensity, near‐threshold exercise.

### 2.3 Pimonidazole administration and tissue collection

Renal ischemia was identified by pimonidazole adduct staining. Pimonidazole (Hypoxyprobe‐1 Omni Kit, HP3‐100KIT, Hypoxyprobe, Inc., Burlington, MA) was administered intraperitoneally at 60 mg/kg immediately after treadmill running. Kidneys were harvested 80 min later and fixed in neutral‐buffered formalin.

### 2.4 In vivo bioluminescence imaging (IVIS) of renal hypoxia

To assess renal hypoxia in vivo, ODD‐Luc mice received D‐luciferin (XLF‐1, Summit Pharmaceuticals International, Tokyo, Japan) at 150 μg/g body weight intraperitoneally 10 min before imaging. Four groups were studied (*n* = 4 per group): Saline, Vasopressin, Treadmill + Vasopressin, and Hypoxia (8% O₂; positive control for 24 h) (Safran et al., 2006). Arginine vasopressin (Sigma‐Aldrich, St. Louis, MO; V9879) was administered intraperitoneally at 0.03 μg/g body weight either immediately after running (Treadmill + Vasopressin) or at the corresponding time without running (Vasopressin).

Images were acquired with an in vivo imaging system (IVIS) Lumina (PerkinElmer, Waltham, MA) under identical settings: exposure 60 s, field of view 12.5 cm, 2× binning, open filter. Mice were imaged from the dorsal side to capture renal signals. Grayscale reference images were overlaid with pseudo‐colored bioluminescence as described (Ushimoto et al., 2023). Background signal intensities at baseline were comparable among groups; therefore, raw photon flux (photons/s) was used without normalization. ROI definition and IVIS statistics. The statistical unit was the individual mouse. To obtain one bilateral ROI per mouse, total flux from right and left kidneys was averaged, (Right + Left)/2. Values were referenced to the baseline before the experiment (“Before Experiment”), and 0 h—the start of the experiment—occurred 24 h after the “Before Experiment” D‐luciferin injection. Groups were as follows: Vasopressin, intravenous vasopressin; Treadmill + Vasopressin, treadmill running (22 m/min for 1 min) immediately before intravenous vasopressin; Hypoxia, exposure to 8% O_2_; Saline, intravenous saline. The primary analysis compared the 0–24 h change across the four groups using one‐way ANOVA. A prespecified planned contrast tested Hypoxia (8% O_2_) versus the mean of Saline, Vasopressin, and Treadmill + Vasopressin. As a nonparametric sensitivity analysis, the Kruskal–Wallis test was also performed.

### 2.5 Immunohistochemistry for AVPR1A and pimonidazole

Paraffin sections (1.0 μm for AVPR1A and 4.0 μm for pimonidazole) were deparaffinized in xylene (10 min × 3), followed by 100% ethanol (10 min × 2), 90% ethanol (10 min), and 80% ethanol (10 min), then rinsed in PBS (5 min × 3). Antigen retrieval was performed in citrate buffer at 95°C–99°C for 30 min, followed by slow cooling to room temperature (~60 min) and PBS washes (5 min × 3). Endogenous peroxidase was quenched with 3% H_2_O_2_, and nonspecific binding was blocked with Protein Block Serum‐Free (X0909, DAKO, Carpinteria, CA). For AVPR1A detection, rabbit anti‐AVPR1A/V1aR primary antibody (ab187753, Abcam, Cambridge, UK) was applied overnight at 4°C; normal rabbit IgG (30000‐0‐AP, Proteintech, Rosemont, IL) served as the isotype control. Secondary detection used Histofine Simple Stain MAX‐PO(R) (414341, Nichirei Bioscience, Tokyo, Japan) for 30 min at room temperature. Signals were visualized with ImmPACT DAB (SK4105, Vector Laboratories, Newark, CA) and counterstained with hematoxylin. For pimonidazole adducts, rabbit anti‐pimonidazole antibody (Ab2627AP, Hypoxyprobe‐1 Omni Kit, Hypoxyprobe, Inc., Burlington, MA) was used (Nakayama et al., 2013) with normal rabbit IgG control (30000‐0‐AP, Proteintech). Primary incubation was 60 min at room temperature, followed by HRP‐linked anti‐rabbit IgG secondary (Cell Signaling Technology, Danvers, MA; #7074S) for 20 min. Visualization and counterstaining were as above.

### 2.6 Human kidney samples

In this study, human tissue samples were collected from regions located at a sufficient distance from the tumor tissue following nephrectomy for renal cell carcinoma. Informed consent was obtained using the opt‐out approach, which had been approved by the ethical review board of Kanazawa Medical University. This study was approved by the ethical review board of Kanazawa Medical University (Kanazawa Medical University Hospital Medical Research Ethics Committee; approved No. I493). This study was also conducted in compliance with the principles outlined in the Declaration of Helsinki (2013).

### 2.7 Reverse‐transcription PCR (RT‐PCR) for AVPR1A

AVPR1A mRNA in kidney cortex, renal artery, and liver of ODD‐Luc mice was assessed by RT‐PCR. Renal arteries were dissected under a stereomicroscope. Total RNA was isolated using RNeasy Mini Plus (74134, Qiagen, Hilden, Germany). cDNA was synthesized with SuperScript IV VILO Master Mix (Invitrogen, 11756050, Thermo Fisher Scientific, Waltham, MA). TaqMan probes were AVPR1A (Mm00444092, Thermo Fisher Scientific) and GAPDH (Mm99999915, Thermo Fisher Scientific). Reactions were run on QuantStudio 3 (Thermo Fisher Scientific). Gene expression was normalized to GAPDH using the ΔΔCt method.

### 2.8 Continuous blood‐pressure monitoring and renal vascular resistance

Mean arterial blood pressure (MBP), mean renal venous pressure (RVP), and renal arterial blood flow (laser Doppler; mL/min/100 g equivalents) (RBF) were measured. Renal vascular resistance (RVR) = (MBP − RVP)/RBF. MBP, RVP, and RBF were recorded for 300 s approximately 80 min after treadmill running.

Six‐ to eight‐week‐old male FVB/NJcl mice were assigned to two groups (*n* = 10 per group): Vasopressin and Control. After the treadmill bout (22 m/min, 1 min), mice were anesthetized with urethane (1.2 g/kg, intraperitoneally) and intubated to maintain stable physiology. Spontaneous respiration was supported with oxygen‐enriched air via a polyethylene endotracheal tube; rectal temperature was maintained at 36.0 ± 0.5°C with feedback control. Polyethylene catheters were placed in the left carotid artery and right jugular vein to measure MBP and RVP, respectively. A catheter was inserted into the left femoral vein for drug or saline administration.

The left renal artery was exposed via a retroperitoneal approach under urethane anesthesia while maintaining normothermia and tissue moisture. A laser‐Doppler tissue blood‐flow meter (OMEGAFLO FLO‐C1; Omegawave, Tokyo, Japan) with a fiber‐optic probe was positioned toward the adventitial surface of the left renal artery using a micromanipulator and stabilized without compressing the vessel. Signals were sampled at 40 Hz and expressed as mL/min/100 g (equivalent units) according to manufacturer calibration, reflecting red‐blood‐cell flux/velocity within the arterial sampling volume. Arterial diameter was not measured. MBP (carotid) and RVP (jugular/renal vein) were recorded simultaneously for subsequent RVR calculation. Pressure signals were transduced and amplified (BP Amp, ADInstruments, Castle Hill, Australia); flow signals were digitized with PowerLab (ADInstruments) at 40 Hz. The zero reference was set at the right atrial level (Mukai et al., 2018).

### 2.9 Drug administration

Arginine vasopressin (0.03 μg/g body weight; Sigma‐Aldrich, V9879) or an equal volume of saline was administered intravenously in the experiment of continuous blood‐pressure monitoring and renal vascular resistance. Data were analyzed offline after completion.

### 2.10 Pharmacological AVPR1A antagonism

To test AVPR1A involvement, the selective non‐peptide antagonist SR49059 (Sigma‐Aldrich, St. Louis, MO) was administered intravenously at 10 μg/g body weight immediately before vasopressin. Mice were assigned to two groups (*n* = 5 per group): Treadmill + Vasopressin with SR49059 or vehicle. Cardiovascular recordings and analyses were performed as above. Animals were euthanized by cervical dislocation under a combination anesthetic.

### 2.11 Whole‐kidney pimonidazole quantification (Figure 3) and statistics

For whole‐kidney quantification, the renal parenchyma was delineated freehand on each section in WinROOF (ver. 2021; Mitani Corporation, Tokyo, Japan) to generate one ROI per kidney.

Primary histology endpoint. A one‐sided Mann–Whitney *U* test (*α* = 0.05) with the directional alternative Treadmill + Vasopressin > Vasopressin was prespecified based on independent renal blood‐flow data. One‐sided 95% lower confidence bounds for the mean difference (Δ = Treadmill + Vasopressin − Vasopressin) were reported using Welch's method and a nonparametric bootstrap; an exact one‐sided permutation test was performed as a robustness check.

### 2.12 Statistics

#### 2.12.1 Figures 2 and 5 (time‐course outcomes)

Renal hemodynamic variables (MBP, RBF, and RVR) were analyzed using two‐way repeated‐measures ANOVA with factors group (between‐subjects) and time (within‐subjects). Post hoc pairwise comparisons at each time point were conducted using Fisher's least significant difference (LSD), conditional on a significant omnibus interaction.

#### 2.12.2 Figure 3 (histology endpoint)

The primary analysis was a prespecified one‐sided Mann–Whitney *U* test (*α* = 0.05; directional alternative Treadmill + Vasopressin > Vasopressin), based on independent hemodynamic findings (Figure 2). Robustness was assessed using a one‐sided exact permutation test and one‐sided 95% lower confidence bounds for the mean difference (Δ = Treadmill + Vasopressin − Vasopressin) obtained by Welch's method and nonparametric bootstrap.

#### 2.12.3 Figure 4 (in vivo bioluminescence)

For each mouse, total flux from the right and left kidneys was averaged to obtain a bilateral ROI [(Right + Left)/2]. Values were analyzed as changes from baseline to 0 h and 24 h. The 0–24 h change was compared across groups using one‐way ANOVA, followed by pairwise post hoc comparisons with Bonferroni correction (Hypoxia vs. the mean of Saline, Vasopressin, and Treadmill + Vasopressin). As a nonparametric sensitivity analysis, a Kruskal–Wallis test was also performed. Pairwise post hoc comparisons with Bonferroni correction were conducted, which showed significant differences between Hypoxia and Treadmill + Vasopressin.

## 3 RESULTS

### 3.1 AVPR1A is expressed in renal vascular smooth muscle in mouse and human kidney

**FIGURE 1:**
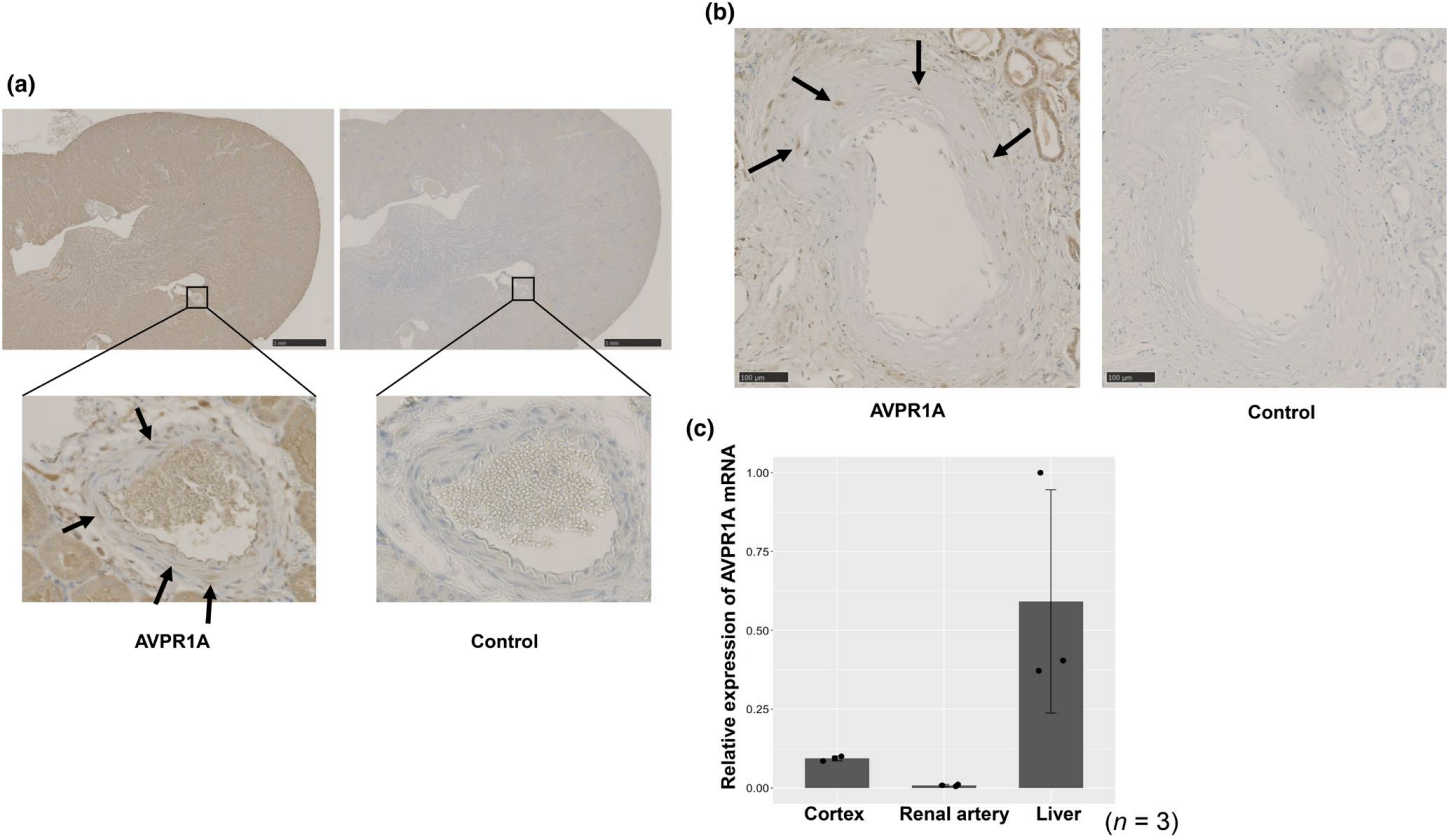
Detection of arginine vasopressin receptor 1A (AVPR1A) expression in mouse and human kidneys. (a) Representative immunohistochemistry (IHC) image showing AVPR1A expression in mouse renal vascular smooth muscle cells (arrows). Scale bar = 1 mm. (b) Representative image from one human kidney sample showing AVPR1A expression in renal vessels. Negative control staining with rabbit IgG showed no positive signal. Scale bar = 100 μm. (c) Quantitative RT‐PCR analysis of AVPR1A mRNA expression in mouse tissues. AVPR1A transcripts were detected in the liver (positive control), renal cortex, and renal artery (*n* = 3). Data are mean ± SEM (*n* = 3).

Immunohistochemistry detected AVPR1A in vascular smooth muscle cells of mouse intrarenal vessels (Figure 1a) and human renal vessels (representative image from one of the human kidney samples in Figure 1b). By quantitative RT‐PCR in mice, AVPR1A mRNA levels were highest in liver, followed by renal cortex and renal artery (Figure 1c).

### 3.2 Treadmill priming augments vasopressin‐induced renal vasoconstriction

**FIGURE 2:**
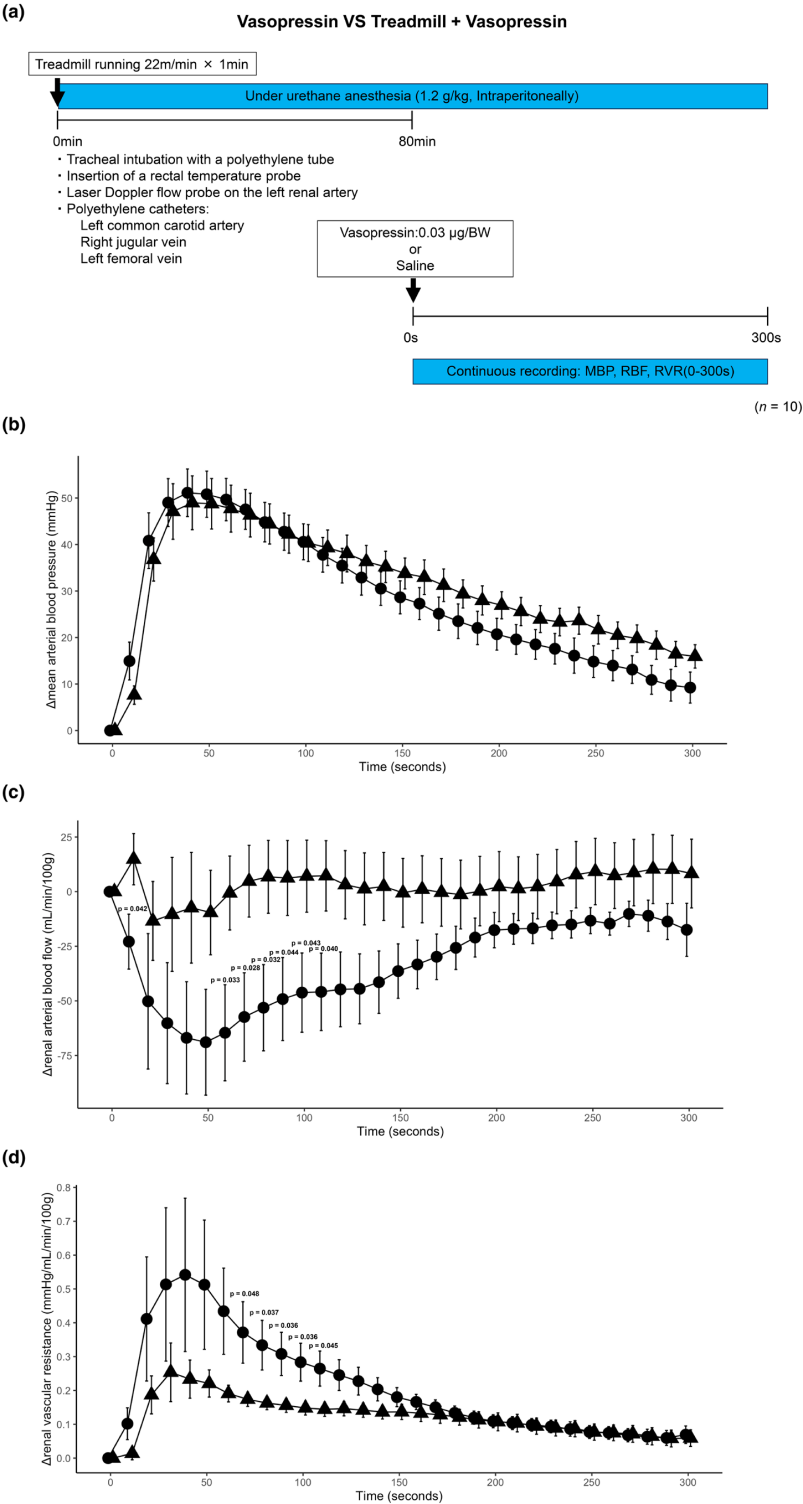
Hemodynamic effects of treadmill and vasopressin. Six‐ to eight‐week‐old male FVB/NJcl mice were subjected to treadmill running (22 m/min, 1 min) or control, followed by intravenous vasopressin (0.03 μg/g body weight) or saline (*n* = 10/group). Continuous hemodynamic monitoring included mean arterial blood pressure (MBP; mmHg), renal arterial blood flow (RBF, laser Doppler; mL/min/100 g), and renal vascular resistance (RVR = (MBP − RVP)/RBF; mmHg/mL/min/100 g). (a) Schematic of the experimental protocol. (b–d) Time‐course changes (Δ from pre‐injection baseline at 0 s) in MBP (b), RBF (c), and RVR (d). Data are expressed as mean ± SEM. Statistical analysis was performed using two‐way repeated‐measures ANOVA with factors group (between‐subjects) and time (within‐subjects), followed by Fisher's LSD for post hoc comparisons. Exact *p* values for significant between‐group differences are shown on the graphs; non‐significant differences are left blank.

The experimental protocol is illustrated in Figure 2a. Time‐course changes in MBP, RBF, and RVR after vasopressin administration (*t* = 0 s) are shown in Figure 2b–d for the Vasopressin and Treadmill + Vasopressin groups. MBP rose in both groups by ~50 mmHg at ~40 s and then declined toward ~20 mmHg at 300 s, with no between‐group differences at any time point (Figure 2b). In contrast, RBF fell rapidly in Treadmill + Vasopressin, but only slightly in Vasopressin. The mean drop in Treadmill + Vasopressin was ~65 mL/min/100 g at ~50 s; pairwise differences were significant at 10 s and from 60 to 110 s (Figure 2c). RVR increased in both groups and was higher in Treadmill + Vasopressin, with significant between‐group differences from 70 to 110 s (Figure 2d). Two‐way ANOVA revealed significant effects of group and time for MBP (group, *p* = 0.002; time, *p* < 0.001), RBF (group, *p* < 0.001; time, *p* < 0.001), and RVR (group, *p* < 0.001; time, *p* < 0.001), whereas the group × time interactions were not significant for any variable (MBP, *p* = 0.963; RBF, *p* = 0.236; RVR, *p* = 0.236). Post hoc Fisher's LSD confirmed the pairwise differences described above.

### 3.3 Treadmill + vasopressin produces corticomedullary‐predominant hypoxia by pimonidazole staining

**FIGURE 3:**
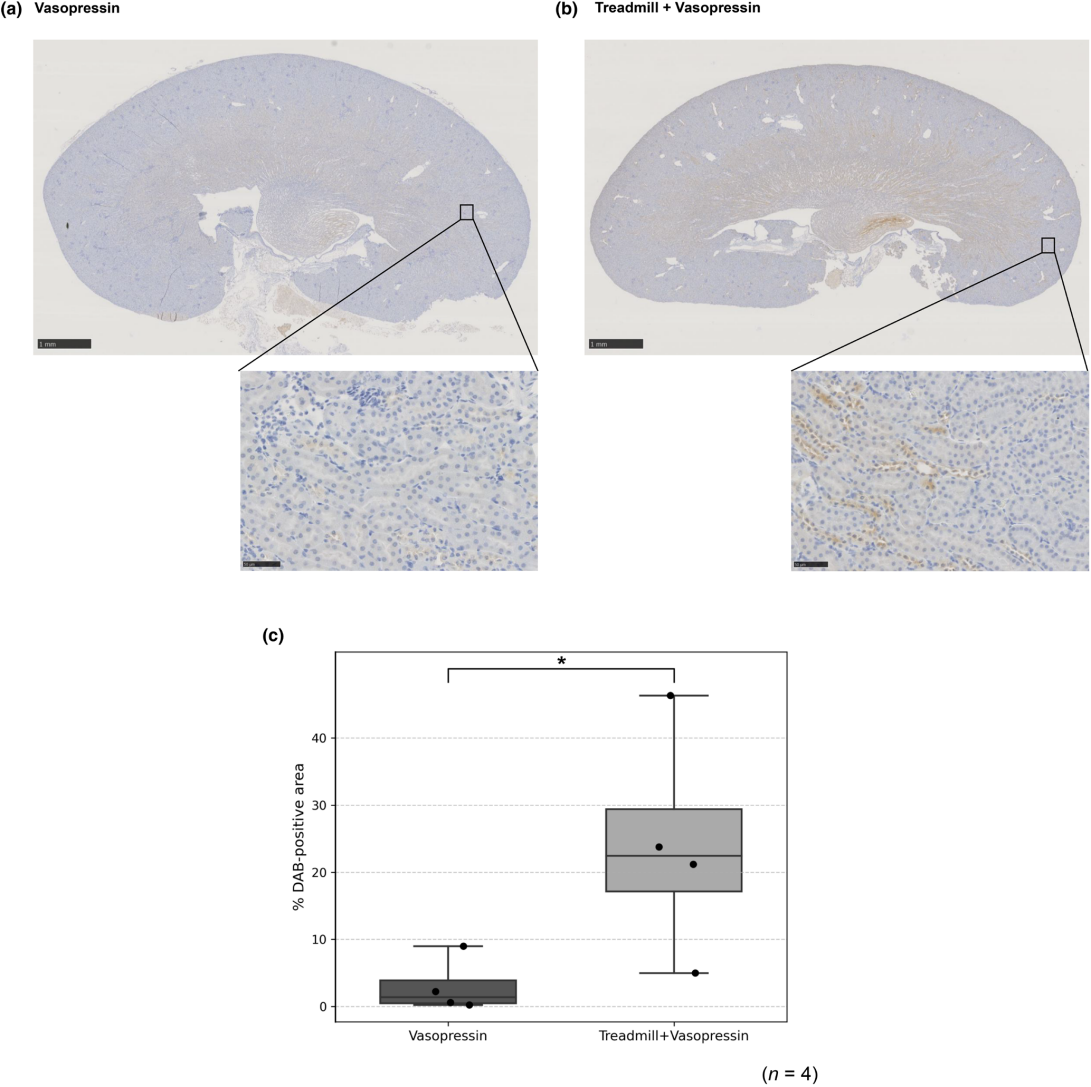
Corticomedullary‐predominant hypoxia after treadmill priming. Paraffin‐embedded kidney sections (4.0 μm) were stained for pimonidazole to detect tissue hypoxia. Representative DAB images show minimal staining in the Vasopressin group (a) and stronger corticomedullary junction (CMJ)‐predominant staining in the Treadmill + Vasopressin group (b). Both panels include low‐magnification (scale bar = 1 mm) and high‐magnification insets (scale bar = 50 μm). (c) Quantification of hypoxic area: Whole‐kidney parenchymal regions of interest (ROIs) were delineated freehand, and % DAB‐positive area was computed (*n* = 4/group). Treadmill + Vasopressin showed significantly greater hypoxia than Vasopressin (mean ± SEM, 24.05 ± 3.12% vs. 2.99 ± 0.91%; one‐sided Mann–Whitney *U* test, *p* = 0.029).

Representative DAB images showed stronger pimonidazole staining in Treadmill + Vasopressin, particularly at the corticomedullary junction, with minimal staining in Vasopressin (Figure 3a,b). Whole‐kidney quantification (freehand parenchymal ROI; manual thresholding) demonstrated a higher % DAB‐positive area with treadmill priming (means 24.05% vs. 2.99%; *n* = 4 per group). The prespecified one‐sided Mann–Whitney *U* test was significant (*p* = 0.029), as was the one‐sided exact permutation test (*p* = 0.029). One‐sided 95% lower confidence bounds for the mean difference (Δ = Treadmill + Vasopressin − Vasopressin) were +1.31 percentage points (Welch) and +8.77 percentage points (bootstrap), both >0, supporting the directional hypothesis (Figure 3c).

### 3.4 Prolonged renal hypoxia by IVIS bioluminescence after treadmill + vasopressin

**FIGURE 4:**
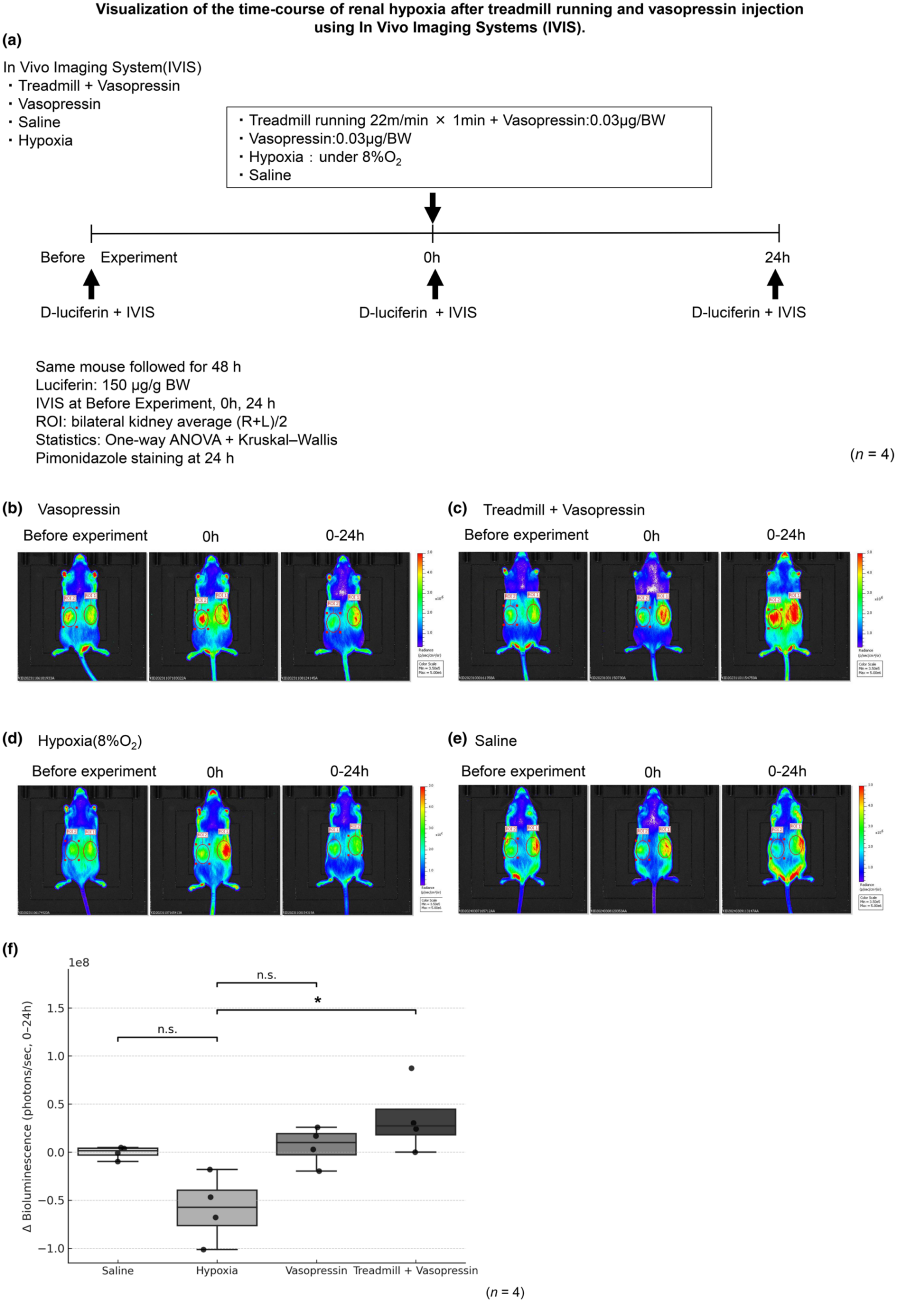
Prolonged renal hypoxia assessed by IVIS bioluminescence after treadmill + vasopressin. ODD‐Luc mice were assigned to four groups (*n* = 4 each): Saline, Vasopressin, Treadmill + Vasopressin, and Hypoxia (8% O_2_, positive control). Mice received D‐luciferin intraperitoneally (150 μg/g body weight) before imaging. Images were acquired with the IVIS Lumina system under identical settings (exposure 60 s, FOV 12.5 cm, 2× binning, open filter). (a) IVIS of the experimental protocol. (b‐e) Representative bioluminescence images at baseline, Experiment (0 h), and 24 h in each group. (f) Quantification of bilateral kidney ROI (mean total flux, photons/sec). Values represent Δ0–24 h change (mean ± SEM). Group differences were assessed by one‐way ANOVA (*p* = 0.003), confirmed by Kruskal–Wallis test (*p* = 0.021). Post hoc Bonferroni‐corrected comparisons showed a significant difference between Hypoxia and Treadmill + Vasopressin (*p* = 0.031), but not between Hypoxia and Vasopressin (*p* = 0.076) or Hypoxia and Saline (*p* = 0.130). *p < 0.05.

Renal hypoxia dynamics were assessed in vivo in ODD‐Luc mice (Figure 4b–e). We defined Before Experiment (baseline, pre‐experiment), Experiment (0 h; initiation of vasopressin and/or treadmill or hypoxia exposure), and 24 h. Bioluminescence (HIF1A‐linked) increased in Treadmill + Vasopressin at Experiment (0 h) and remained prominent at 24 h; a similar early increase was observed in Vasopressin at 0 h. By 24 h, signals returned to near‐baseline in Hypoxia, whereas Treadmill + Vasopressin remained elevated, indicating prolonged hypoxia. No material change was observed in Saline.

For the interval‐coded bilateral ROI, the 0–24 h change differed among groups (one‐way ANOVA, *p* = 0.003), confirmed by the nonparametric Kruskal–Wallis test (*p* = 0.021). Post hoc pairwise comparisons with Bonferroni correction (Hypoxia vs. each group) revealed that Treadmill + Vasopressin exhibited significantly greater sustained luminescence than Hypoxia (*p* = 0.031). In contrast, Hypoxia did not differ significantly from Vasopressin (*p* = 0.076) or Saline (*p* = 0.130). These findings indicate that treadmill priming delayed recovery from vasopressin‐induced renal hypoxia.

### 3.5 AVPR1A antagonism abrogates vasopressin effects after treadmill priming

**FIGURE 5:**
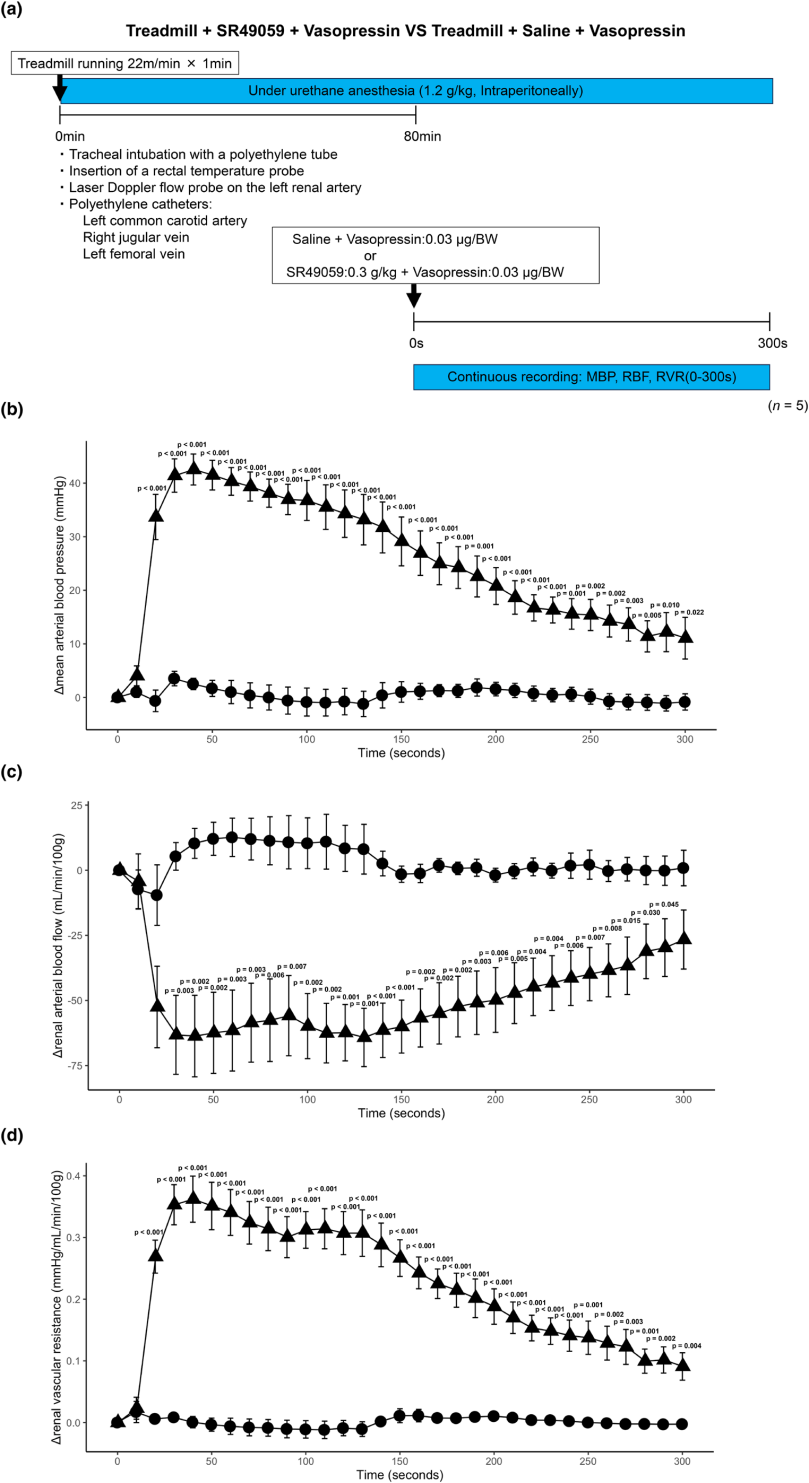
Hemodynamic effects of AVPR1A antagonist (SR49059). Six‐ to eight‐week‐old male FVB/NJcl mice were subjected to treadmill running (22 m/min, 1 min) followed by intravenous vasopressin (0.03 μg/g body weight) with or without the selective AVPR1A antagonist SR49059 (10 mg/kg, *n* = 10/group). Continuous hemodynamic monitoring included mean arterial blood pressure (MBP; mmHg), renal arterial blood flow (RBF, laser Doppler; mL/min/100 g), and renal vascular resistance (RVR = (MBP − RVP)/RBF; mmHg/mL/min/100 g). (a) Schematic of the experimental protocol. (b–d) Time‐course changes (Δ from pre‐injection baseline at 0 s) in MBP (b), RBF (c), and RVR (d). Data are expressed as mean ± SEM. Statistical analysis was performed using two‐way repeated‐measures ANOVA with factors group (between‐subjects) and time (within‐subjects), followed by Fisher's LSD for post hoc comparisons. Exact *p* values for significant between‐group differences are shown on the graphs; nonsignificant differences are left blank.

To test receptor specificity, we administered the selective AVPR1A antagonist SR49059 prior to vasopressin in treadmill‐primed mice (experimental protocol shown in Figure 5a). Compared with Treadmill + Saline + Vasopressin, SR49059 abolished or markedly attenuated the MBP rise and the divergent RBF/RVR responses (Figure 5b–d). Two‐way ANOVA revealed significant effects of group and time for MBP (group, *p* < 0.001; time, *p* < 0.001), as well as a significant group × time interaction (*p* < 0.001). For RBF, the group effect (*p* < 0.001) and the group × time interaction (*p* = 0.017) were significant, whereas the time effect was not (*p* = 0.920). For RVR, significant effects were observed for group (*p* < 0.001), time (*p* < 0.001), and group × time interaction (*p* < 0.001). Post hoc Fisher's LSD confirmed significant between‐group differences for MBP (20–300 s), RBF (30–290 s), and RVR (20–300 s).

## 4 DISCUSSION

In this study, vasopressin administration after brief high‐intensity treadmill running resulted in a greater reduction in renal arterial blood flow (RBF), an increase in renal vascular resistance (RVR), and renal hypoxia compared with vasopressin alone, indicating that the post‐exercise state enhances AVPR1A‐dependent vasoconstriction. These effects were prevented by the selective AVPR1A antagonist SR49059, supporting an AVPR1A‐mediated vasoconstrictive mechanism. Hypoxia was demonstrated both spatially—by pimonidazole staining centered at the corticomedullary junction (CMJ)—and temporally—by in vivo bioluminescence imaging showing sustained signals beyond 24 h. Thus, our data establish sufficiency and receptor specificity (treadmill priming + exogenous AVP; blockade by SR49059). Importantly, we did not directly test exercise‐induced changes in AVPR1A expression, localization, trafficking, or downstream signaling.

### 4.1 Pattern of hypoxia and species differences

Pimonidazole staining in Treadmill + Vasopressin revealed CMJ‐predominant, widespread hypoxia, whereas Vasopressin alone showed minimal staining. This pattern differs from the wedge‐shaped, patchy delayed contrast washout on contrast‐enhanced CT in patients with ALPE ~24 h after contrast administration. A plausible explanation is renal lobulation: mice have monolobular kidneys, whereas humans are multilobular (Zhou et al., 2023). Heterogeneous lobar perfusion in multilobular kidneys can yield wedge‐shaped changes, while monolobular mouse kidneys manifest more global, CMJ‐weighted hypoxia. These anatomical differences likely contribute to the phenotypically different ischemic distributions across species. Previous studies have implicated vasopressin in AKI across exercise‐related and other contexts (Chapman et al., 2019; García‐Arroyo et al., 2016, 2017; Hodgson et al., 2017). Vasopressin gene expression and processing have also been characterized (Ivell et al., 1986), and collecting duct cells can synthesize vasopressin in response to hypertonicity (Arroyo et al., 2022). However, it has not been directly demonstrated whether acute exercise can sensitize the kidney to vasopressin‐induced hypoperfusion and hypoxia. In this study, we show for the first time that treadmill exercise markedly enhances AVPR1A‐dependent renal vasoconstriction and sustained hypoxia, as demonstrated by real‐time hemodynamic measurements and multimodal imaging (IVIS and pimonidazole).

### 4.2 Time course and mechanistic interpretation

By IVIS, the Hypoxia (8% O_2_) positive control showed a steep rise at Experiment (0 h) with a decline over 24 h. In contrast, Vasopressin and Treadmill + Vasopressin exhibited more protracted elevation from 24 h, indicating delayed resolution. Together with the hemodynamic data, these findings indicate that acute hypoxic stress can trigger ischemia, whereas exercise‐related vasopressin signaling contributes to its prolongation. We did not quantify AVPR1A transcripts or protein after the 22 m/min, 1‐min bout and therefore do not claim receptor upregulation by exercise. Because the exaggerated responses emerge within seconds to minutes, functional sensitization of renal resistance vessels (e.g., sympathetic co‐activation, downstream signaling) is a more plausible explanation than receptor upregulation.

### 4.3 Therapeutic implications

SR49059 abolished or markedly attenuated the vasopressin effects after treadmill priming, consistent with AVPR1A's acute vasoconstrictor and inotropic roles (Hupf et al., 1999; Szczepanska‐Sadowska, 2022) and its longer‐term mitogenic actions (Szczepanska‐Sadowska, 2022). Beyond acute hemodynamics, AVPR blockade has shown benefit in cardiorenal contexts (Ikeda et al., 2015; Lemmens‐Gruber & Kamyar, 2006). Within the kidney, AVPR1A contributes to acid–base handling via intercalated cells (Giesecke et al., 2019), and AVPR1A antagonists have demonstrated renoprotective effects in chronic kidney disease (CKD) models (Okada et al., 1995), including reduced proteinuria and vascular injury in partial‐nephrectomy, high‐salt, hypertensive rats (Okada et al., 1995), potentially via effects on the renin–angiotensin–aldosterone system and adrenal stress hormones (Lebedeva et al., 2023). Collectively, our data support AVPR1A antagonism as a candidate strategy for ALPE prevention or treatment, while acknowledging that clinical translation requires targeted trials.

### 4.4 Limitations

This study has several limitations. First, the relatively small sample size in certain experimental groups (e.g., *n* = 4) is a limitation of this study. Although statistically significant differences were detected, the small sample size may reduce the power to detect smaller or more subtle effects. Future studies with larger cohorts and prospective power analyses are warranted to confirm and extend these findings. Second, we did not directly measure circulating vasopressin levels. In mice, vasopressin secretion is pulsatile and short‐lived, and repeated blood sampling under anesthesia and vascular cannulation could themselves alter hormonal dynamics. Therefore, we adopted a pharmacological strategy, combining exogenous vasopressin administration with selective AVPR1A antagonism, to establish receptor‐specific causality without direct hormone assays. Third, we did not include a separate exercise‐only group. However, since vasopressin was administered after treadmill running and physiological parameters were continuously monitored before vasopressin administration, we were able to assess the effects of exercise alone during the pre‐vasopressin period. No significant changes in renal blood flow or vascular resistance were observed immediately after treadmill running, suggesting that exercise alone did not cause renal hypoxia or hemodynamic instability. Therefore, we believe that the inclusion of a separate exercise‐only group may not be essential for interpreting the primary outcomes of this study. Fourth, measurements were restricted to male mice and to laser‐Doppler signals from a localized arterial sampling volume, which reflect RBC flux rather than absolute volumetric flow. Finally, pimonidazole quantification relied on manually delineated parenchymal ROIs and a prespecified global threshold; automated pipelines could further reduce measurement variability.

## 5 CONCLUSIONS AND PROPOSED MODEL

**FIGURE 6:**
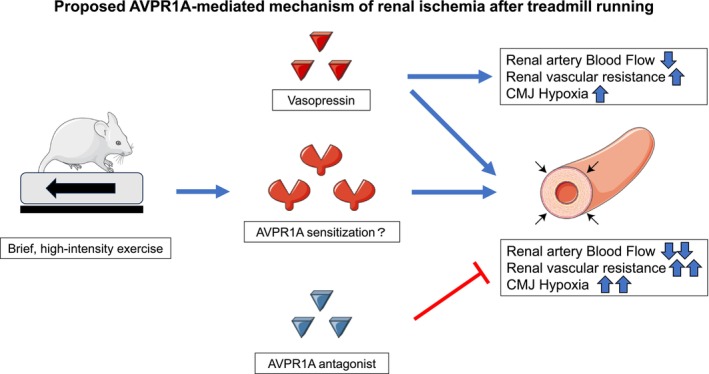
Proposed mechanism of AVPR1A‐mediated renal ischemia after treadmill exercise. Schematic summary: Brief high‐intensity treadmill running primes renal resistance vessels. Subsequent vasopressin activates AVPR1A in vascular smooth muscle, leading to exaggerated vasoconstriction, reduced renal arterial blood flow, increased vascular resistance, and sustained corticomedullary‐predominant hypoxia. SR49059 blocks these effects, confirming AVPR1A involvement. Some elements were adapted from Servier Medical Art (https://smart.servier.com), licensed under a Creative Commons Attribution 3.0 Unported License.

We show that brief high‐intensity exercise followed by exogenous AVP elicits AVPR1A‐dependent renal vasoconstriction and prolonged CMJ‐predominant hypoxia, which is blocked by SR49059—establishing sufficiency and receptor specificity. We hypothesize, but do not demonstrate, that exercise may transiently “prime” renal resistance vessels to vasopressin via AVPR1A sensitization. A proposed (hypothesized) model is provided in Figure 6, illustrating how treadmill running could enhance renal vascular responsiveness to vasopressin, leading to exaggerated vasoconstriction, reduced RBF, and sustained renal hypoxia.

## AUTHOR CONTRIBUTIONS

Kazutoshi Nomura drafted the manuscript. Kazutoshi Nomura edited and revised the manuscript. Takao Iwawaki provided the mice used in the experiments. Kazutoshi Nomura and Mamoru Tanida conducted the renal blood flow measurement experiments, under the guidance of Mamoru Tanida and Yasutaka Kurata. Kazutoshi Nomura conducted the IVIS experiments, with guidance from Takao Iwawaki and Ryoko Akai. Statistical analysis was performed by Kazutoshi Nomura and Norifumi Hayashi, under the guidance of Keiji Fujimoto. Tomohisa Yabe, Ai Fujii, Kanae Nomura, Keiichiro Okada, Kazuaki Okino, Norifumi Hayashi, and Keiji Fujimoto provided advice for the experiments. Kengo Furuichi supervised the overall project.

## FUNDING INFORMATION

This study was supported by a grant from the JSPS KAKENHI Grant number JP20H04133 and JP24K11398.

## CONFLICTS OF INTEREST STATEMENT

The authors declare no conflicts of interest associated with this manuscript.

## ETHICS STATEMENT

All procedures were approved by the Kanazawa Medical University Animal Experimentation Committee (Approval No. 2020‐34) and conducted in accordance with institutional guidelines. Mice were euthanized by cervical dislocation under a combination anesthetic according to facility practice.

## DISCLAIMERS

The views and opinions expressed in this dissertation/thesis are solely those of the author and do not necessarily reflect the official policies or positions of Kanazawa Medical University or the advisory committee.

## Data Availability

The datasets generated and/or analyzed during the current study are available from the corresponding author on reasonable request.
